# Supplementary material for: Functional Evolution of Pseudofabraea citricarpa as an Adaptation to Temperature Change
Source: J Fungi (Basel). 2024 Jan 28;10(2):109. doi: 10.3390/jof10020109 (PMC10890082; doi:10.3390/jof10020109)
Supplement: Supplementary file 1 [file jof-10-00109-s001.zip › jof-2770688-supplementary/jof-2770688-Supplementary-Figures.pdf]

Functional evolution of *Pseudofabraea citricarpa* as an adaptation to climate change

LIU Sai-fei[1,†], CHEN Li[2,†], QIAO Xing-hua[2], REN Jie-qun[3], ZHOU Chang-yong[4,*], YANG Yu-heng[1,*]

[1] *Key Laboratory of Agricultural Biosafety and Green Production of Upper Yangtze River (Ministry of Education), College of Plant Protection, Southwest University, Beibei, Chongqing, 400716, China*

[2] *Plant Protection and Fruit Tree Technology Extension Station of Wanzhou District in Chongqing, 404199, China*

[3] *The Chongqing Three Gorges Academy of Agricultural Sciences, 404150, China*

[4] *Citrus Research Institute, Southwest University, Beibei, Chongqing, 400712, China*

* *Correspondence: zhoucy@cric.cn (C.Z.); yyh023@swu.edu.cn (Y.Y.)*

† *These authors contributed equally to this work.*

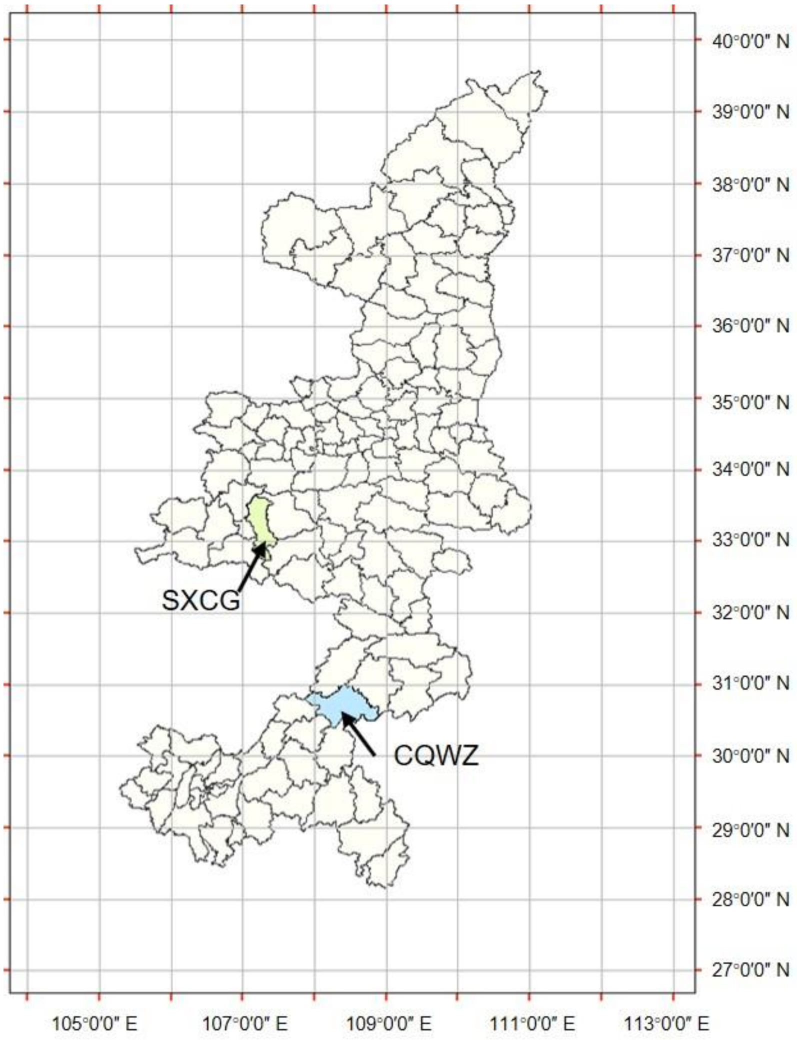

Figure S1. Geographical location of *Pseudofabraea citricarpa* sampling.

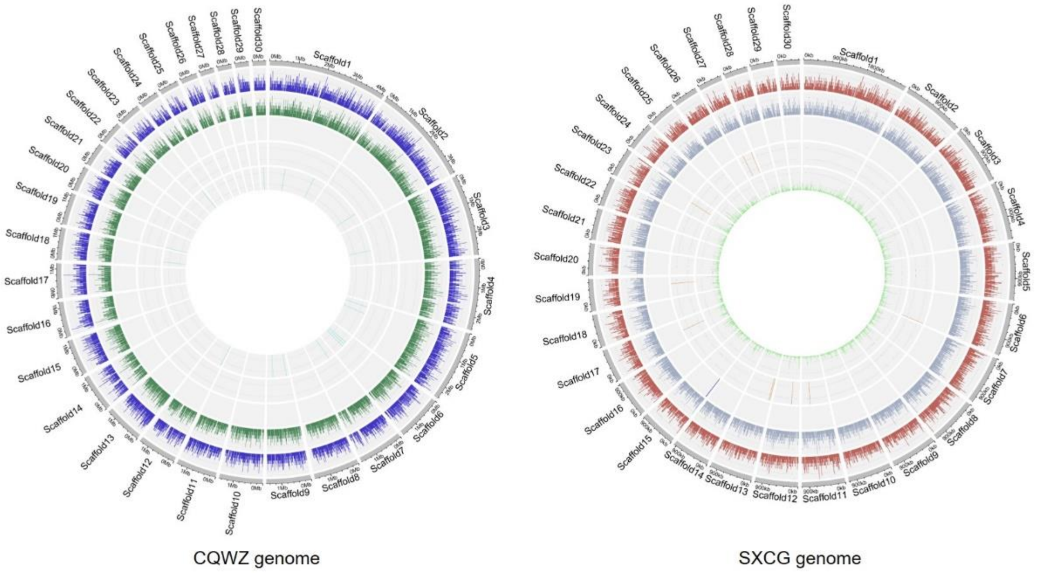

Figure S2. Genome assembly mapping of *Pseudofabraea citricarpa*.

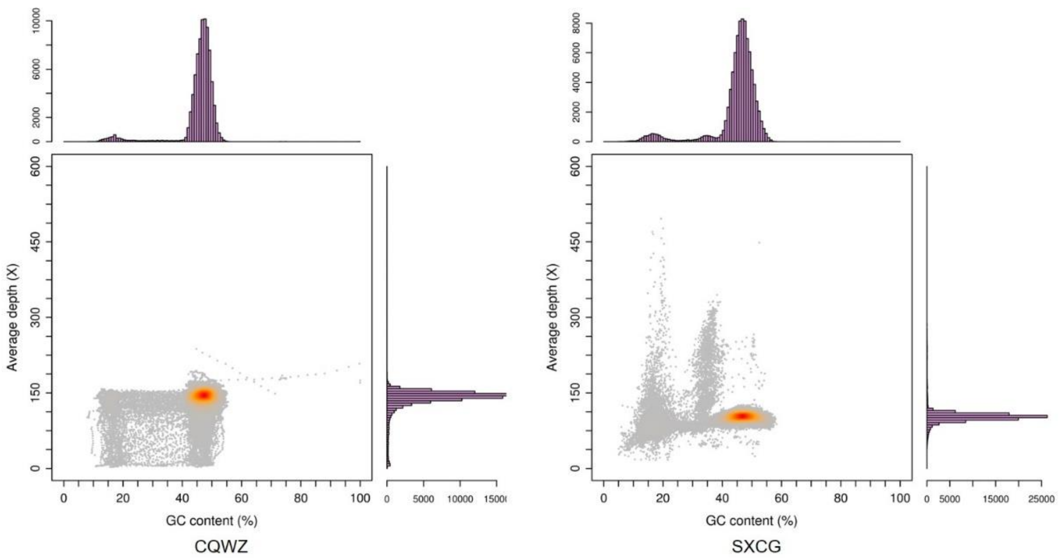

Figure S3. GC depth distribution analysis of *Pseudofabraea citricarpa***.**

The original reads from the PE library were aligned to the assembled sequence using SOAP software to obtain base coverage depth. A window of a chosen size was selected to move along the sequence base by base (or non-repetitive base by base), and the average depth and GC content were calculated for each window. This information was used to generate a GC-depth plot.

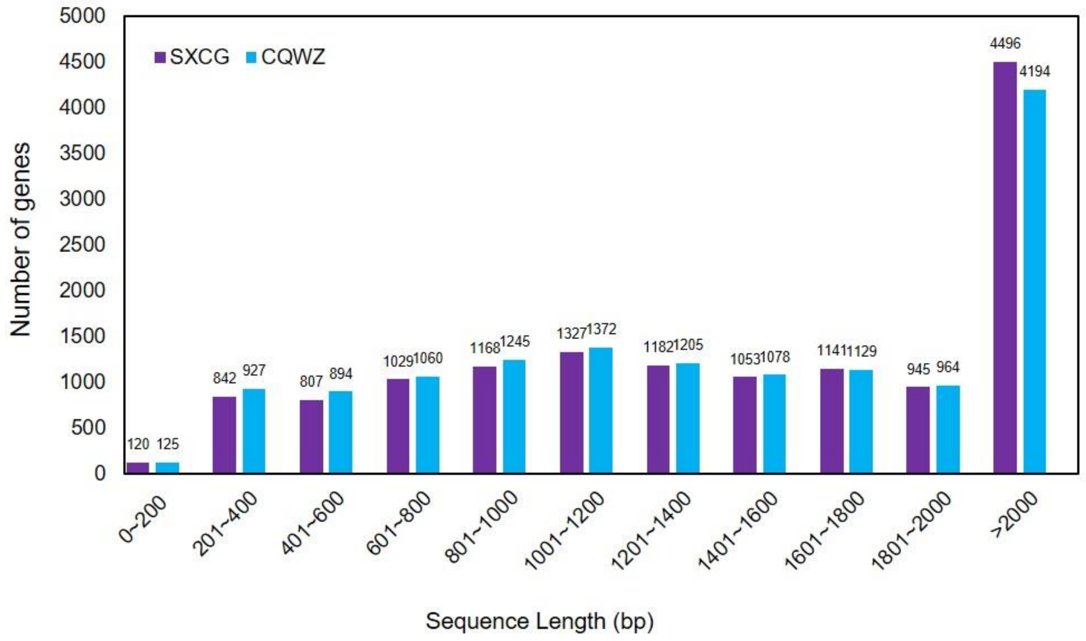

Figure S4. Length of genes encoded in the genome of *Pseudofabraea citricarpa*.

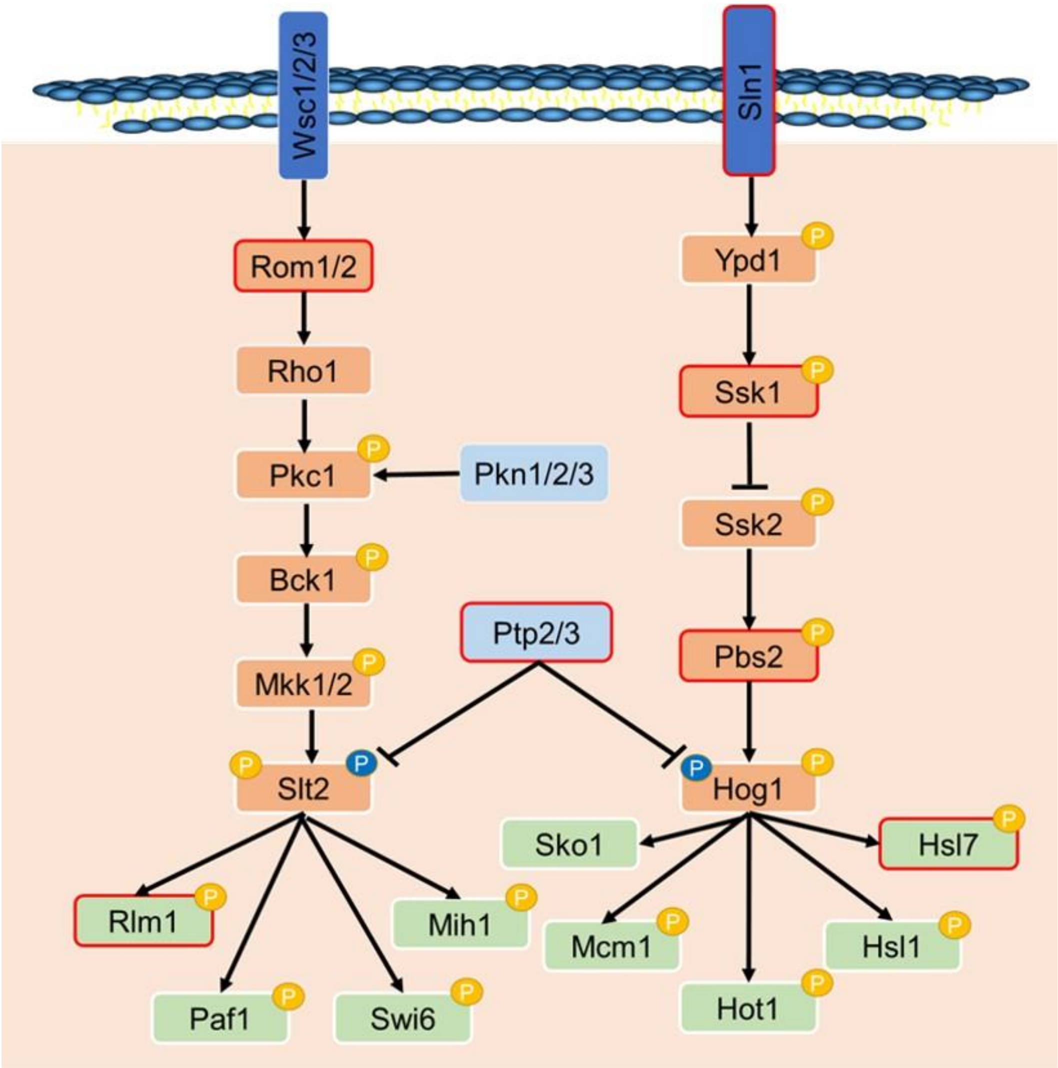

Figure S5. Differences between the two strains in the MAPK pathway.

Red boxes indicate amino acid sequence differences.

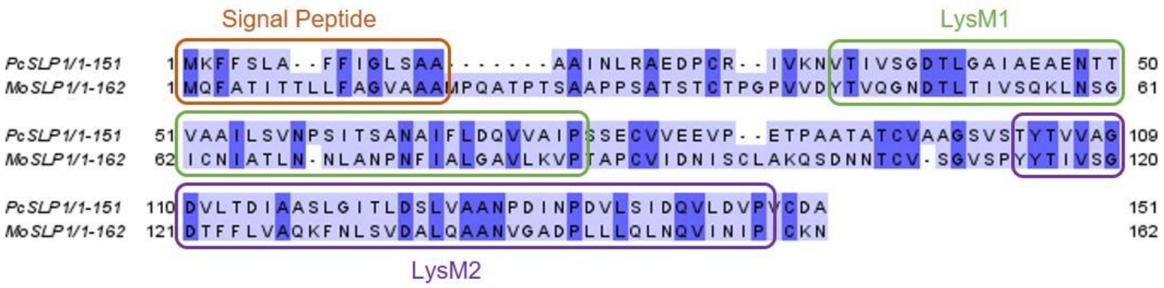

Figure S6. Sequence analysis of LysM effector SLP1.

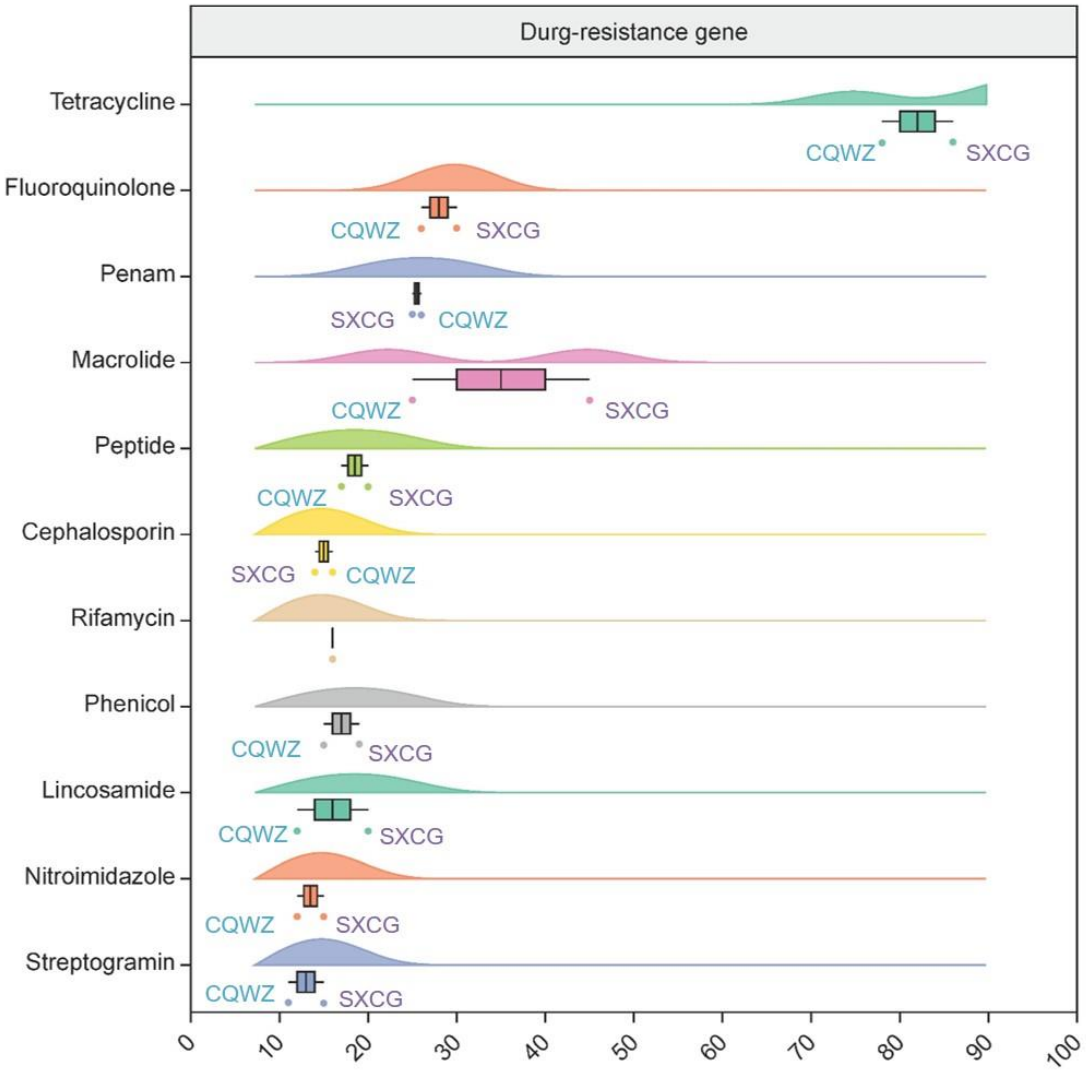

Figure S7. Differences in the number of resistance genes between the SXCG and CQWZ.
